# The Role of Circulating MicroRNAs in the Prediction of Response to Neoadjuvant Chemotherapy in Locally Advanced Breast Cancer in the Indian Population

**DOI:** 10.7759/cureus.59553

**Published:** 2024-05-02

**Authors:** Shyam Tripathi, Jayanthi Mathaiyan, Smita Kayal, Rajesh Nachiappa Ganesh

**Affiliations:** 1 Department of Pharmacology, Jawaharlal Institute of Postgraduate Medical Education and Research, Puducherry, IND; 2 Department of Medical Oncology, Jawaharlal Institute of Postgraduate Medical Education and Research, Puducherry, IND; 3 Department of Pathology, Jawaharlal Institute of Postgraduate Medical Education and Research, Puducherry, IND

**Keywords:** rt-pcr, pbmc, labc, nac, mirna

## Abstract

Introduction: MicroRNAs (miRNAs) are known to play an important role in cancer cell proliferation, susceptibility of cancer cells to chemotherapy, and patient survival. Identifying miRNAs that can predict response to chemotherapy in locally advanced breast cancer (LABC), the most common variant, can help to choose appropriate drug regimens to suit the epigenetic profile of individual patients.

Objective: To investigate the expression of the differentially expressed miRNAs identified by next-generation sequencing from a pilot study involving cases and controls, in peripheral blood mononuclear cells (PBMC) of patients with LABC during the course of neoadjuvant chemotherapy (NAC) and determine their role in response to chemotherapy.

Methods: This study included 30 newly diagnosed LABC patients. Peripheral blood from every participant was collected before the start of chemotherapy, at the end of the third cycle, and at the end of the seventh cycle of NAC. Based on the results of a pilot study in a similar population with suitable controls, four differentially expressed miRNAs namely miR-24-2, miR-192-5p, miR-3609, and miR-664b-3p were considered to be validated in this study. The expression of these four miRNAs was examined by qRT-PCR, and their association with response to chemotherapy was analyzed.

Result: A significant change in the expression of miR-192-5p was found in responders (p = 0.001) over a period of seven cycles and the difference between the expression of miR-24-2 from baseline to the seventh cycle of NAC was higher in responders while compared to the non-responders (p < 0.05).

Conclusion: miR-192-5p and miR-24-2 were identified as predictive biomarkers for response to NAC in south Indian patients with LABC.

## Introduction

Breast cancer (BC) is the most common cancer in women, with an overall worldwide prevalence of 2.3 million cases which account for 11.7% of all cancers. It is the fifth leading cause of cancer mortality with 685,000 deaths worldwide [1]. In India, BC accounts for 13.5% of total cancers and 26.3% of cancer in women [2]. Late diagnosis is common, where 50-60% were reported to have locally advanced breast cancer (LABC) at presentation to the hospital [3,4]. The standard treatment for LABC is neoadjuvant chemotherapy (NAC) prior to surgical removal of the tumor. One of the established NAC regimens involves patients receiving three cycles of 5-fluorouracil (500 mg/m^2^), epirubicin (100 mg/^2^), cyclophosphamide (500 mg/m^2^) (FEC) followed by four cycles of Docetaxel (75 mg/m^2^) [5]. Based on a literature search, only 8-31% of patients achieved a pathological complete response rate (pCR) (no residual tumor being detected in the post-operative histopathological examination) and the rest are exposed to harmful effects of therapy without much benefit [6-9]. BC prognosis is traditionally based on tumor, node, and metastasis (TNM) staging, molecular subtype, and BRCA1 gene family mutation. Currently, accurate evaluation of tumor response to NAC is limited, and measurement of conventional BC markers like ER, PR, P-53, carcinoembryonic antigen (CEA), cancer antigen 15-3 (CA15-3) & HER-2/neu has shown limited predictive value in the NAC setting [9,10].

MicroRNAs (miRNAs) are a naturally occurring class of short non-coding RNA molecules, 19 to 25 nucleotides in length, known to regulate gene expression at the post-transcriptional level [11]. They are known to work in one network, where a change in the expression of one miRNA causes a chain reaction involving many genes from the same or distinct pathway [12]. More than 2000 human miRNAs have been identified and believed to modify nearly one-third of human coding genes [13]. miRNAs are known to play a role in a variety of processes like development, differentiation, proliferation, and apoptosis, as well as in the formation of cancer [14]. Nearly half of the human miRNAs are found in cancer-associated genomic regions that are frequently amplified, deleted, or altered in cancer, implying that some of the miRNAs may operate as tumor suppressor miRNAs (suppression of several biomolecules involved in malignancies) or onco-miRNAs (activate cancer-related signaling pathway) [15,16].

miRNAs appear to be passively released by dying cells, and hence circulating miRNA profiling could be representative of the response to anticancer therapy. Dysregulation of miRNA expression and its function in the evolution of breast carcinoma was initially characterized in 2005. Since then, many miRNAs with aberrant expression in connection with BC progression have been found [17]. Some of the studies have reported that the expression levels of miRNAs were directly associated with the initiation, development, and manifestation of BC and its prognosis and treatment at the cellular and subcellular levels [18,19]. Earlier, we analyzed blood samples from 15 cases (women with LABC who were treatment naïve) and 15 controls (healthy aged-matched women). Four miRNAs (miR-24-2, miR-192-5p, miR-3609, and miR-664b-3p) were found to be differentially expressed in next-generation sequencing (NGS) [20]. 

miR-192-5p has been reported to act as a tumor suppressor by inhibiting cell proliferation and inducing apoptosis in BC [21]. According to Du et al., miR-24 promotes epidermal growth factor (EGF) expression by targeting phosphatases tyrosine-protein phosphatase non-receptor type 9 (PTPN9) and receptor-type tyrosine-protein phosphatase F (PTPRF) [22]. According to Wu et al, miR-664 acts as a tumor suppressor by suppressing the invasion and proliferation in BC by targeting insulin receptor 1 [23]. An in-vitro study showed that restoration of miR-3609 expression sensitized BC cells to doxorubicin by blocking the PD-L1 immune checkpoint pathways [24].

There are limited studies on the impact of chemotherapy on miRNA expression patterns and the predictive value of circulating miRNA expression profiles have not been studied extensively. The objective of this study is to investigate the expression of four miRNAs in peripheral blood mononuclear cells (PBMC) of women with LABC during treatment with NAC. Therefore, we wanted to validate and evaluate the change in expression of the four miRNAs identified by NGS in the pilot study in a cohort of women diagnosed with LABC and treated with NAC for their association with response to therapy.

## Materials and methods

This was a prospective study conducted at the Regional Cancer Centre of the Jawaharlal Institute of Postgraduate Medical Education and Research, Puducherry, India. The Institutional Ethical Committee of Jawaharlal Institute of Postgraduate Medical Education and Research approved the study (JIP/IEC/2017/267) and written informed consent was obtained from all study participants. Women of South Indian ethnicity who were diagnosed with LABC in the age range of 18-70 were included in the study. Pregnant and lactating women, patients already on treatment, or with triple-negative BC were excluded from the study.

Demographic and clinical data were collected from hospital records. Patients diagnosed with LABC received three cycles of (5-fluorouracil (500 mg/m^2^), epirubicin (100 mg/^2^), cyclophosphamide (500 mg/m^2^)) (FEC) followed by four cycles of Docetaxel (75 mg/m^2^). After seven cycles of NAC, the patient undergoes a modified radical mastectomy. Recruited participants were followed till the postoperative period and the biopsy report was assessed for pathological response to chemotherapy. Based on the histopathology report of the resected surgical specimen, the responder and non-responder were defined as given below.

Responder

A patient whose report showed a complete response (CR) or partial response (PR) was considered to be a responder.

CR was considered when there was no evidence of residual tumor in all resected specimens of breast and axillary lymph nodes and partial response when there was a decrease in cellularity within the tumor or multiple scattered foci in which the presence of the largest foci was considered as the residual tumor.

Non-responder

Patients whose report showed no response were considered non-responders.

No response was considered when the pre-surgical pre-NAC biopsy was reported as invasive breast carcinoma and the post-surgery biopsy was reported as a minimal response with little or no change in the size of the tumor.

RNA isolation and quantification of miRNA by real-time PCR

PBMCs were isolated by density gradient centrifugation using Ficoll-Paque. Diluted blood was layered over the density gradient medium and centrifuged to get a buffy coat which was washed with phosphate-buffered saline (PBS) to obtain PBMCs.

**Figure 1:**
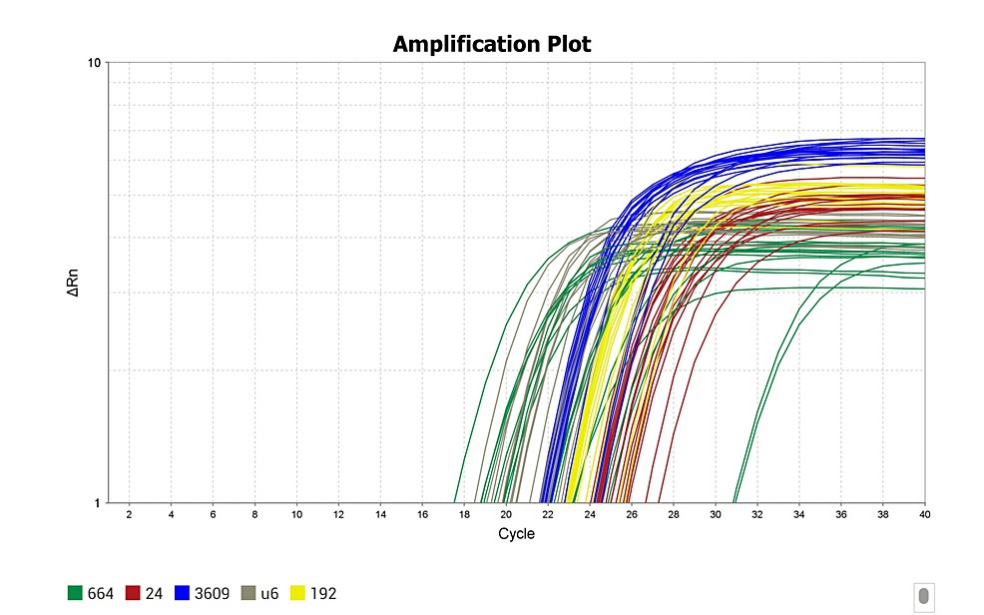
The Ct value for miR-24-2, miR-192-5p, miR-3609 and miR664b-3p and housekeeping gene U6

RNA isolation from PBMCs was done by kit method (R2051, Direct-zol RNA Miniprep, Zymo Research Cooperation, Irvine, United States). Isolated RNA was quantified by spectrophotometer and subjected to cDNA conversion using a kit (miRCURY LNA RT Kit, Cat. No.339340, Qiagen, Hilden, Germany). The 10 µl reverse transcription reaction which contains an RNA template, RT enzyme buffer, and nuclease-free water mixture was incubated for 60 minutes at 42°C and for 5 minutes at 95°C. The converted cDNA was used for the miRNA profile expression by real-time quantitative reverse transcription polymerase chain reaction (Real-time qRT-PCR, Quant studio3 from Applied Biosystems, Waltham, United States). A total of 3 µl of cDNA was used per 10 µl qPCR reaction for expression analysis which includes SYBR green dye and miRNA-specific detection primers. Real-time quantitative reverse transcription polymerase chain reaction (qRT-PCR) was performed at 95°C for 2 minutes and 40 cycles at 95°C for 10 seconds and 56°C for 1 minute followed by a melt curve with three steps, 95°C for 15 seconds, 60°C for 1 minute and 95°C for 15 seconds using RT-PCR (Applied Biosystems). All the samples were run in duplicate and the threshold was determined using the Quant studio software version v1.5.1 (Applied Biosystems, Waltham, United States) and then the average value was determined. U6 was used as a housekeeping gene. The amplification plot for miRNAs and housekeeping gene as reference is shown in Figure 1.

We used 2-Δct to determine the expression level of miRNA where Δct was calculated as the difference between Ct values of the gene of interest and that of the housekeeping gene.

Statistical analysis

Data were recorded and analysed with IBM SPSS Statistics for Windows, Version 22 (Released 2013; IBM Corp., Armonk, New York, United States). In all the patients miRNA was quantified as a relative expression. Friedman’s test was used to analyse the change in miRNA expression at three-time points. The Mann-Whitney U test was used to analyse change in miRNA expression between the responder and non-responder. A p-value of <0.05 was considered statistically significant.

## Results

Characteristics of study participants

Out of 75 patients screened, 30 were included in the study. The demographic and clinical data of these 30 patients are summarized in Table 1. The majority of the patients had estrogen receptor positivity (70%) were luminal B (43%) by molecular classification and had grade 2 cancer (87%). In this study, 73% were responders and 27% were non-responders.

**Table 1:** Baseline and clinical characteristics of the study participants BSA: body surface area

Clinical characteristics	Frequency (%) N = 30
Age	
≤50	14 (47)
>50	16 (53)
BMI	26.02 ± 7.73
BSA	1.63 ± 0.30
Menopausal status	
Pre	13 (43)
Post	17 (57)
Comorbidities	
Diabetes	9 (30)
Hypertension	12 (40)
Ischemic heart disease	01 (3)
Asthma	02 (6)
Thyroid	02 (6)
Receptor status	
ER+	21 (70)
PR+	13 (43)
HER 2+	17 (57)
Molecular subtype	
Luminal A	08(27)
Luminal B	13 (43)
Her 2 enriched	7 (23)
Unknown	02 (7)
Nottingham histological grading	
Grade 1	04 (13)
Grade 2	26 (87)
Grade 3	Nil (0)
Response to chemotherapy	
Responder	22 (73)
Non-responder	08 (27)

Change in miRNA expression in BC patients who received NAC

We evaluated changes in miRNA expression over a period of time in response to seven cycles of NAC in LABC patients. miR-192-5p expression was found to be increased after the third cycle with a subsequent reduction after the seventh cycle. This change in expression was found to be statistically significant by the Friedman test with a p-value of 0.001 only in the responders (Table 2) but not in non-responders (Table 3). For other miRNAs (miR-24-2, miR-3609, and miR-664b-3p), we did not find a significant change.

**Table 2:** miRNA expression at baseline after third and seventh cycles of NAC among responders *p < 0.05. miRNAs: microRNAs; NAC: neoadjuvant chemotherapy; IQR: interquartile range

Sl. No	Type of miRNA	miRNA expression				
		Before NAC median & IQR	After 3rd cycle of NAC Median & IQR	After the 7th cycle of NAC Median & IQR	Test statistic	p-value
1.	miR-24-2	0.0383 (0. 02, 0.12)	0.125 (0.05, 0.19)	0.125 (0.05, 0.29)	4.36	0.11
2.	miR-664b-3p	0.340 (0.07, 0.73)	0.338 (0.03, 0.66)	0.517(0.12, 0.96)	1.455	0.48
3..	miR-3609	0.166 (0.08, 0.38)	0.175 (0.13, 0.30)	0.172 (0.07, 0.54)	2.54	0.28
4.	miR-192-2	0.098 (0.03, 0.26)	0.934 (0.84, 0.98)	0.154 (0.11, 0.28)	32.81	0.001*

**Table 3:** Change in miRNA expression at baseline after third and seventh cycles of NAC among non-responders miRNAs: microRNAs; NAC: neoadjuvant chemotherapy; IQR: interquartile range

S.no	Type of miRNA	miRNA expression			
		Before chemo median & IQR	After 3rd cycle of chemo median & IQR	After 7 cycles of chemo median & IQR	p-value
1.	miR-24-2	0.046 (0.01, 0.36)	0.088 (0.04, 0.39)	0.031 (0.01, 0.14)	0.32
2.	miR-192-2	0.080 (0.02, 0.54)	0.946 (0.7, 0.99)	0.078 (0.04, 0.29)	0.09
3.	miR-3609	0.261 (0.09, 0.31)	0.462 (0.06, 0.84)	0.059 (0.01, 0.29)	0.42
4.	miR-664b-3p	0.487 (0.21, 0.75)	0.405 (0.04, 0.88)	0.132 (0.03, 1.42)	0.41

We further analyzed the difference in miRNA expression between baseline and after seven cycles of chemotherapy between the responders and non-responders. miR-24-2 expression was found to be statistically significant between the two groups with a p-value of 0.027 (Table 4) by the Mann-Whitney U test.

**Table 4:** Comparison of change in miRNA expression from baseline to completion of NAC between responders and non-responders *p < 0.05. miRNAs: microRNAs; NAC: neoadjuvant chemotherapy; IQR: interquartile range

Types of mi-RNA	miRNA expression		
	Responder (n = 22) median & IQR	Non-responder (n = 8) median & IQR	p-value
miR-24-2-5p	0.086 (-0.02, 0.17)	-0.018 (-0.23, 0.01)	0.027*
miR-192-2-5p	0.039 (-0.08, 0.25)	-0.005 (-0.40, 0.03)	0.20
miR-3609	0.031 (-0.25, 0.21)	0.103 (-0.09, 0.25)	0.62
miR-664b-3p	-0.002 (-0.40, 0.62)	-0.165 (-0.43, 0.71)	0.59

## Discussion

miRNAs, a class of short non-coding RNA molecules play a crucial role in regulating gene expression and are engaged in various biological activities, such as cell division, proliferation, and programmed cell death. In the context of cancer, miRNAs assume dual roles. Some act as tumor suppressors, while others function as oncogenes. Dysregulation of miRNA expression is associated with the development and progression of cancer. Therefore, understanding the role of miRNAs in cancer pathogenesis is essential for developing new diagnostic and therapeutic approaches.

We assessed the expression of four miRNAs (miR-24-2, miR-192-5p, miR-3609, and miR-664-3b-5p) at three time points in 30 LABC patients who received NAC. Our study found that the expression of miR-192-5p was increased after three cycles of FEC in the responders, indicating that it could have a tumor suppressor function or its activity is enhanced by FEC. Moreover, miR-192-5p could have sensitized the responders to the effect of chemotherapy with 5-fluorouracil, epirubicin, and cyclophosphamide. We did not find any study that reported the alteration of miR-192-5p expression in response to NAC in LABC patients. Hence we have compared our findings with studies that reported the expression of these miRNAs on cancer patients. Similar to the findings of our study, another study reported lower expression of miR-192-5p in blood samples of Caucasian women with BC in comparison to healthy women [25]. miR-192-5p expression was downregulated in the tumor tissue samples of Iranian women with BC when compared to adjacent non-cancerous tissue [26]. Two in-vitro studies demonstrated that miR-192-5p inhibits cell proliferation by inhibiting caveolin and enhances apoptosis by down-regulating mdm2 [27,28]. An in-vitro study reported that miR-192-5p sensitized BC cells to doxorubicin-induced apoptosis [21]. Expression of miRNA-192-5p was reported to be downregulated in lung and colon cancers also [29,30]. However miR-192-5p expression was upregulated in cancers like pancreatic, hepatocellular, and gastric [31-33]. These studies indicate that miR-192-5p plays a tumor suppressor role in lung, breast, and colon cancers and has an oncogenic role in pancreatic, hepatocellular, and gastric cancers which needs validation in further studies.

We also analyzed the expression levels of four different miRNAs before and after chemotherapy and found that there was a significant increase of miR-24-2 expression in responders when compared to the non-responders in our study indicating that it could have tumor suppressor function. miR-24-2 was reported to down-regulate Bcl-2, a protein that is involved in regulating apoptosis in MCF-7 BC cell line [34]. Bcl-2 is known to act as proto-oncogenes and contributes to malignancy by protecting cells from apoptosis [35]. We did not find any study that reported the expression of miR-24-2 in blood samples as well as the effect of NAC on this miRNA. In two studies reported from India the expression of miR-24-2 was found to be low in tumor tissue samples of treatment naïve BC patients as compared to normal tissue [34-36]. In addition, it was found that the expression of miRNA-24-2 was decreased as the stage of BC increased indicating its tumor suppressor role in healthy individuals/early stages. This finding is similar to our study where the tumor suppressor role of miR-24-2 was associated with a better response to NAC.

In our study, we used PBMCs for isolation of miRNAs which probably accounted for the similarity in the results with miRNA in breast tissue of LABC patients from other studies. PBMCs are the cells known to be affected early in the development of cancer as well as responses to chemotherapy [37].

In our study, the expression of miR-192-5p and miR-24-2 was altered during FEC treatment. The impact of chemotherapy on miRNA expression is becoming increasingly evident in various cancer types. Anticancer drugs such as 5-FU, capecitabine, epirubicin, bevacizumab, cyclophosphamide, gemcitabine, and fludarabine have been linked to alterations in miRNA profiles. This phenomenon is observed in lung, breast, adenocarcinoma, chronic lymphocytic leukemia, as well as pancreatic and colorectal cancers [38,39]. In a study involving patients undergoing NAC, it was observed that the expression levels of miR-4449, miR-141-3p, miR-4465, and miR-1181 exhibited distinct changes at different time points during chemotherapy in patients who responded positively to the treatment [40]. These findings suggest a possible influence of cancer chemotherapy on miRNA expression. Our study did not find any significant difference in the other two miRNAs (miR-3609, miR-664b-3p) between responders and non-responders. Other in-vitro studies showed that miR-3609 may reverse chemoresistance in BC by blocking the PD-L1 immune checkpoint, suggesting that miR-3609 may have a role in sensitizing BC cells to chemotherapy [22]. High miR-3609 expression is associated with a longer survival rate in TNBC patients where it induced the cell cycle arrest and inhibited TNBC development [41]. miR-664b-3p was found to enhance apoptosis (programmed cell death) by regulating the levels of BRIP1, which is a protein that interacts with BRCA1 (a tumor suppressor gene commonly associated with BC) [42]. A low level of this miRNA was found in cancer patients after chemotherapy as compared to healthy individuals [43]. Further research is needed to fully understand the roles of miR-3609 and miR-664b-3p in cancer progression and response to therapy.

The limitation of this study was that we did not have a control group and did not find expression of miRNAs in the tumor tissue. However, the four miRNAs studied here were based on NGS of samples from both cases and controls. Moreover, we used PBMCs in blood for miRNA expression which are known to have higher concentrations of miRNAs.

## Conclusions

The findings of our study indicate that miR-192-5p and miR-24-2 have a role in tumor suppression in LABC patients who are on NACT. The identification of patients with lower expression of these miRNAs may prompt the consideration of alternative therapeutic strategies, underscoring the importance of personalized medicine in the management of LABC. Furthermore, our study did not observe a significant change in the expression levels of miR-3609 and miR-664b-3p between responders and non-responders. This information adds to our understanding of how different miRNAs play roles in responding to BC treatment.
